# Hypothalamic Cavernous Angioma Associated With Memory and Behavior Disturbance Attacks: Role of Imaging in Diagnosis

**DOI:** 10.5812/iranjradiol.6737

**Published:** 2012-03-25

**Authors:** Preetam Bhujagonda Patil, Muralidhar Gururajrao Kamalapur, Jayaraj Chandrashekhar Sindhur, Shyamsundar Krishnabhat Joshi

**Affiliations:** 1 Department of Radiodiagnosis and Imaging, S.D.M College of Medical Sciences and Hospital Dharwad, Dharwad, India; 2 Department of Medicine, S.D.M College of Medical Sciences and Hospital Dharwad, Dharwad, India

**Keywords:** Hemangioma, Cavernous, Hypothalamus, Magnetic Resonance Imaging

## Abstract

Deep seated cavernous angioma (CA) is a very rare entity, those occurring in the hypothalamus are still less common. We present a case of a 52-year-old man who presented with behavior and memory disturbance attacks. He had a CA of the hypothalamus as revealed by magnetic resonance imaging (MRI). We will discuss the role and importance of imaging in such scenario and also the differential diagnoses of this rare entity.

## 1. Introduction 

Cavernous angioma (CA) is a congenital vascular malformation consisting of thin walled sinusoidal spaces lined with epithelium without interposing glial or neural tissue [1]. The incidence of CA is 0.3% to 0.7% in the general population representing 10%-20% of all vascular malformations. They mostly occur in the supratentorial compartment (80%), followed by the infratentorial compartment (15%) and the spinal cord (5%). Cavernous malformations of the optic pathway and hypothalamus are extremely rare. They represent 1% or less of all cavernous malformations. In the study of 65 cases of cavernous malformation involving the optic pathway and the hypothalamus by Liu et al., only five were found to be in the hypothalamus and none of these patients presented with psychological complaints [2]. We report a rare case of CA in the hypothalamus who was associated with memory and behavior disturbance which to the best of our knowledge has not been reported before.

## 2. Case Presentation 

A 52-year-old man recently detected with diabetes presented with brief episodes of anger and irrelevant talk from one month ago, each episode lasted for about 2-3 minutes followed by return to the normal state in approximately fifteen to twenty minutes. Patient had 3-4 episodes in a month. The patient also had transient loss of memory and altered bowel habits from one month ago and was evaluated for altered bowel habits with a stool test and colonoscopy, which were normal.

General central nervous system (CNS) examination was normal. The patient was referred to the radiology department for computed tomography (CT) scan.

**Figure 1:**
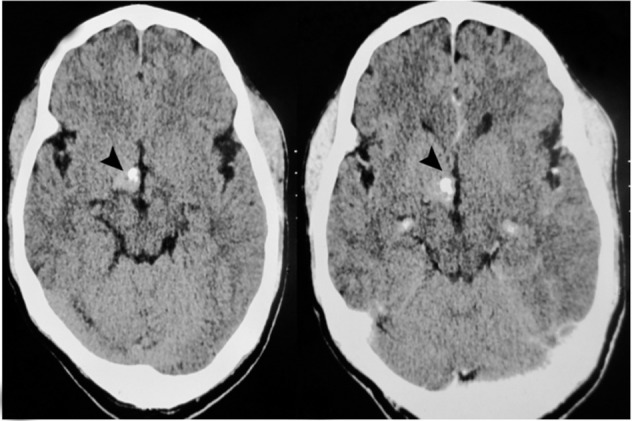
A, Axial plain; and B, Post contrast show a mildly enhancing hyperdense nodular mass on the right side of the hypothalamus with punctate calcification (arrow head) without surrounding edema or mass effect.

**Figure 2:**
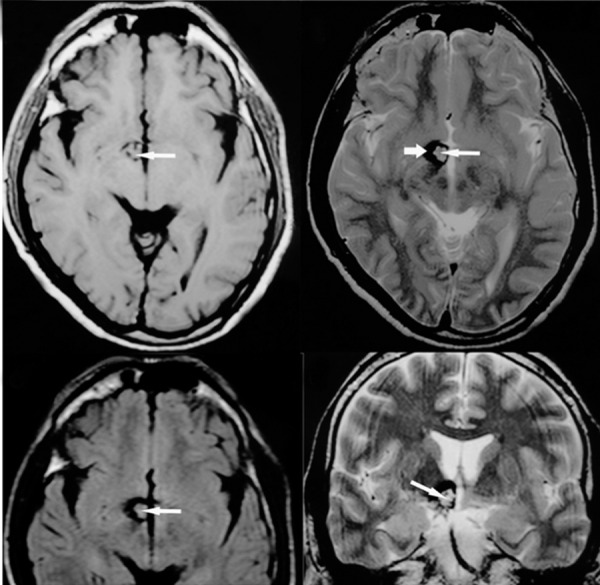
A, Axial T1 weighted; B, Axial T2 weighted; C, Axial FLAIR; and D, Coronal T2 weighted images show a small well-defined mass on the right side of the hypothalamus with a mixed intensity core appearing like “popcorn” (long arrow) surrounded by a low signal intensity hemosiderin rim (short arrow) seen on T2 weighted images with no edema or mass effect as noted on FLAIR images.

CT scan showed a mildly enhancing hyperdense nodular mass on the right side of the hypothalamus, 8-10 mm in diameter with punctate calcification without surrounding edema or mass effect (Figure 1A-B). A provisional diagnosis of CA was made based on the presence of a hyperdense mass with calcification showing mild enhancement on post contrast study. The patient was referred to MRI for confirmation. Axial T1 weighted magnetic resonance imaging (MRI) (Figure 2A); axial T2 weighted (Figure 2B); axial fluid attenuated inversion recovery (FLAIR) (Figure 2C); and coronal T2 weighted images (Figure 2D) showed a small well-defined mass on the right side of the hypothalamus with a mixed intensity core appearing like “popcorn”. The mass was surrounded by a low signal intensity hemosiderin rim seen on T2 weighted images. There was no edema or mass effect as noted on FLAIR images.

Presence of mixed intensity core with a hypointense rim noted on T2 weighted images were the basis on which a diagnosis of CA was made.

Patient and family members were counseled for further neurosurgical intervention as the lesion was deep seated, but the patient refused any further treatment. The patient was further advised to come for follow up scans for which he refused and he now continues to have behavioral problems.

## 3. Discussion 

Vascular malformations in CNS are divided into four groups; namely, arteriovenous, venous, capillary telangiectasis and cavernomas [3]. Cavernoma (cavernous hemangiomas, angiomas, cavernous malformation) is a benign vascular lesion occurring at any site within the CNS.

The etiology of CA is not known. A small number of cases are hereditary with high penetrance and autosomal dominant transmission. Recently, a gene has been mapped to chromosome 7q11-q22 in Hispanic and some white people, but not in all families. The hereditary form is highly prevalent in Hispanic and North Americans. CA is assumed to be congenital as they do not display any neoplastic features [4]. A correlation between radiation and cavernomas of the brain has been determined, particularly in case of a positive history of radiation exposure in childhood [5].

Macroscopically, these lesions resemble a ripe mulberry and microscopically they contain blood filled cavities lined by a single layer of endothelium and separated by neuroglia without any neural tissue [6].

CT demonstrates these lesions as focal high attenuation masses with variable calcification in the absence of edema or mass effect. Enhancement is typically mild on CT which may not be identifiable [7].

MRI is reliable in the detection, follow up and diagnosis of symptomatic and asymptomatic CA. CA appear as a reticulated core of mixed signal representing blood in various stages of degeneration surrounded by a hypointense halo due to hemosiderin on T2W MRI. T1W image also demonstrates a mixed signal core, but is less sensitive. Some cases show contrast enhancement, but it is usually not marked and the mass effect is noted after acute bleeding [4]. High-field MRI is highly specific in the diagnosis of CA which obviates the need for pathological confirmation [1][7].

In our case, MRI revealed a well-defined lesion with a central focus of reticulated high signal intensity surrounded by a rim of signal void which is characteristic of CA. We do not have the pathological confirmation as the patient refused surgery.

CA varies in size from a few millimeters to a few centimeters. The majority of them are small, but may reach a significant size. Those measuring more than 6 cm in diameter are termed as giant CA [8]. Patients with CA are often asymptomatic and when symptoms are present, they depend on the location and size of the lesion. Most frequent presentations are focal neurological deficits, hemorrhage and epilepsy [9].

Our case presented with brief episodes of abnormal behavior and transient memory loss which is an uncommon symptom associated with CA. The patient probably had an autonomic disturbance as evidenced by gastrointestinal motility dysfunction. The patient and family members were counseled for gamma knife radiosurgery as the lesion was deep seated, but the patient refused any further treatment. He was further advised to come for follow-up scans, but he refused and he continues to have behavioral problems. It is probable that the abnormal clinical findings of the current case have occurred due to CA; thus, our case indicates that chronic compression and hemorrhage could cause behavioral and memory disturbance which is an uncommon presentation of CA. Microsurgical removal of superficial and deep seated CA which present with hemorrhage, neurological deficits and seizures have good prognosis with minimal risk of complications. Gamma knife radiosurgery is another option in cases of inaccessible and deep seated CA [10].

In a study by Liu et al. on 65 cases of cavernous malformations involving the optic pathway and hypothalamus, only five cases were found in the hypothalamus. These patients presented with chief complaints of acute visual loss, headache, unsteady gait, convulsions, progressive visual loss, dementia and lower limb weakness. Of these five patients, one underwent subtotal resection whose outcome was stable, remaining four who underwent gross total resection out of which two improved and the other two were stable [2]. In all the previously reported cases, the patients presented without behavioral symptoms; CA in this case was associated with memory loss and behavioral change.

MRI findings are diagnostic of CA which usually do not require any further confirmation. By any means; however, in very rare cases of ambiguity, some of the differential diagnoses which may be thought of are hypothalamic glioma, germinoma, hamartoma, histiocytosis and granuloma, but presence of mixed intensity “popcorn” like core and a hypointense hemosiderin rim helps in ruling out these conditions.

Lesions in the fronto-temporal region are a common cause of behavioral and memory disturbances, However lesions at rare locations like the hypothalamus may also manifest with memory and behavioral disturbances and CA is a rare cause to present with these symptoms, but should be considered as one of the possibilities. CA can be effectively diagnosed on MRI, based on the presence of a reticulated core of mixed signal representing blood in various stages of degeneration surrounded by a hypointense halo due to hemosiderin on T2 weighted images. These characteristic CA findings help in the differential diagnosis and also obviate the need for pathological confirmation.
